# Global Immune-Nutrition-Inflammation Index as a novel comprehensive biomarker in predicting the radiation-induced trismus rates in locally advanced nasopharyngeal carcinoma patients

**DOI:** 10.17305/bb.2024.10616

**Published:** 2024-12-01

**Authors:** Efsun Somay, Erkan Topkan, Sibel Bascil, Nilüfer Kılıc Durankuş, Şükran Senyürek, Düriye Ozturk, Berrin Pehlivan, Ugur Selek

**Affiliations:** 1 Department of Oral and Maxillofacial Surgery, Faculty of Dentistry, Baskent University, Ankara, Turkey; 2 Department of Radiation Oncology, Faculty of Medicine, Baskent University, Adana, Turkey; 3 Department of Periodontology, Faculty of Dentistry, Baskent University, Ankara, Turkey; 4 Department of Radiation Oncology, School of Medicine, Koc University, Istanbul, Turkey; 5 Department of Radiation Oncology, Faculty of Medicine, Afyonkarahisar Health Sciences University, Afyonkarahisar, Turkey; 6 Department of Radiation Oncology, Bahcesehir University, Istanbul, Turkey

**Keywords:** Radiation-induced trismus (RIT), Global Immune-Nutrition-Inflammation Index (GINI), concurrent chemoradiotherapy (CCRT), nasopharyngeal carcinoma

## Abstract

Nasopharyngeal carcinoma (NPC) is a prevalent cancer in certain regions, often treated with concurrent chemoradiotherapy (CCRT), which can lead to complications such as radiation-induced trismus (RIT). In this study, we aimed to evaluate whether the novel pretreatment Global Immune-Nutrition-Inflammation Index (GINI) can predict RIT in locally advanced nasopharyngeal carcinoma (LA-NPC) patients undergoing CCRT. Data of LA-NPC patients presenting without RIT were reviewed retrospectively. Any post-CCRT maximum mouth openings (MMO) ≤ 35 mm were considered RIT. The GINI index was calculated using the formula: GINI ═ (CRP × Monocytes × Platelets × Neutrophils) ÷ (Albumin × Lymphocytes). We used receiver operating characteristic (ROC) curve analysis to examine the potential correlation between pretreatment GINI measures and post-CCRT RIT status. Logistic regression analysis examined the independence of the association between confounding factors and RIT rates. The study comprised 230 participants, and 52 (22.6%) received an RIT diagnosis. The optimal pre-CCRT GINI cutoff that dichotomizes RIT rates was determined to be 1424 (area under the curve [AUC]: 76%; sensitivity: 75.0%; specificity: 71.7%; J-index: 0.463). RIT incidence was significantly higher in the GINI ≥ 1424 group than in its GINI < 1424 counterpart (43.3% vs 9.3%; Hazard ratio (HR): 4.76; *P* < 0.001). Multivariate logistic regression analysis revealed that a pre-CCRT GINI ≥ 1424 was an independent predictor of increased RIT rates after definitive CCRT in this patient group (*P* < 0.001). In conclusion, the present results revealed that elevated pre-CCRT GINI measures (≥ 1424) can efficiently and independently predict elevated RIT rates in LA-NPC patients after CCRT.

## Introduction

Radiation therapy (RT) is a vital treatment modality for various head and neck cancers (HNC), including nasopharyngeal cancers (NPC). However, despite its effectiveness, it can bring about complications that can cause significant discomfort and impair the patient’s quality of life [1]. One of these complications is radiation-induced trismus (RIT), a condition characterized by the restriction of mouth opening, jaw stiffness, and pain [2]. RIT can significantly impact the patient’s ability to perform daily activities, such as speaking, swallowing, eating, and performing daily oral hygiene leading to a decline in overall well-being [3]. The incidence of RIT varies significantly depending on the size and location of the tumor, and the literature reports rates ranging from 5% to 42% of HNC patients [4, 5].

High doses of ionizing radiation produce RIT by damaging the muscles, connective tissues, and blood vessels in the masticatory region, resulting in radiation-induced fibrotic tissue repair [6]. Although these changes may lead to RIT development, the underlying mechanisms remain unknown. However, there is a growing interest in using inflammatory biomarkers to diagnose and manage RIT, as it is well recognized that inflammation plays a crucial role in its development and progression [3, 5]. Proinflammatory cytokines, such as transforming growth factor-beta (TGF-β), tumor necrosis factor-alpha (TNF-α), and interleukin-6 (IL-6) are known to contribute to the inflammatory process and promote fibrotic changes in irradiated tissues, thus playing a pivotal role in the genesis of RIT [7]. Previous studies by Somay et al. [8–10] and Topkan et al. [11] have provided compelling data about the impact of inflammatory biomarkers on RIT. They have shown that pre-treatment ratios, such as neutrophil-to-platelet ratio (NLR), hemoglobin-to-platelet ratio (HPR), pan-immune-inflammation value (PIV), and Valero’s Host Index (which includes neutrophils, monocytes, lymphocytes, hemoglobin, and albumin) have a significant impact on the occurrence of RIT [8–11].

The Global Immune-Nutrition-Inflammation Index (GINI) was developed by Topkan et al. [12] and includes neutrophils, monocytes, platelets, lymphocytes, albumin, and C-reactive protein (CRP) in its formula. In their original study, Topkan et al. found that GINI has potential prognostic value for patients with stage IIIC non-small cell lung cancer (NSCLC) undergoing concurrent definitive chemoradiotherapy (CCRT). Based on this evidence and the fact that the GINI index combines multiple immunological, inflammatory, and nutritional biomarkers, we proposed that it may also accurately predict treatment-related toxicities, such as RIT. All of these factors play a crucial role in the initiation and progression of RIT. Therefore, we conducted a retrospective cohort analysis to assess the predictive value of the GINI index for the incidence of RIT in patients with locally advanced nasopharyngeal carcinoma (LA-NPC) undergoing definitive CCRT.

## Materials and methods

### Study population

A retrospective data search was conducted on patients with LA-NPC diagnoses who underwent CCRT and were evaluated at the Radiology, Dentistry, Medical Oncology, and Radiation Oncology Departments of the Baskent University Faculty of Medicine between January 2010 and January 2023. This period was chosen to prevent treatment technique-related biases, as the IMRT option became available at our institution in 2010. To be eligible for the study, patients had to meet the following criteria: Being at least 18 years old, having a confirmed diagnosis of squamous cell NPC, and being classified as LA-NPC (T1-2N1-3M0 or T3-4N0-3M0) based on the AJCC staging framework (8^th^ edition). Additionally, they must not have a previous diagnosis of temporomandibular disorder (TMD) or trismus, based on the current diagnostic criteria (DC) for TMD, known as DC/TMD before undergoing CCRT.

Patients with a previous history of RT or chemotherapy, inadequate cardiac, renal, hepatic, or pulmonary function were not included in this study. The exclusion criteria also applied to those with confirmed active infections, chronic immune or inflammatory disorders, recent use of steroids or antibiotics within the past 30 days, and recent blood transfusions within 90 days, in order to minimize potential interference with the variables being studied. Patients who had undergone surgery for neck trauma, muscle-related pain, myofascial pain syndrome, primary tumors, or lymph node invasion of masticatory muscles were also excluded. In addition, patients being treated for local recurrence, with a history of surgery and/or RT to the head and neck region, and those with a follow-up period of less than six months were not eligible to participate in the study.

### Treatment protocol

Our recommended treatment for LA-NPC is simultaneous integrated boost intensity-modulated radiotherapy (SIB-IMRT). To identify the target volumes for RT, we use pretreatment co-registered computed tomography (CT), 18-fluorodeoxyglucose-positron emission CT (PET-CT), and/or magnetic resonance imaging (MRI) images of the entire neck and the affected nasopharyngeal primary. The current study followed established guidelines, previously documented, to determine target volumes and corresponding dosages for RT [13]. The following is a summary of the doses that were given to the planning target volumes (PTVs): Single daily RT fractions in 33 days (5 days/week) were used to administer total doses of 70.0, 59.4, and 54.0 Gy for high-risk, intermediate-risk, and low-risk PTVs, respectively [13]. Concurrent chemotherapy consisted of weekly cisplatin (40 mg^2^). For those able to tolerate it, two cycles of adjuvant cisplatin and 5-fluorouracil combination (every 21 days) were recommended. Patients received analgesics, antiemetic medications, and nutritional supplements as needed as part of the supportive care protocol.

### Baseline and follow-up oral evaluation and the determination of radiation-induced trismus (RIT)

An experienced oral and maxillofacial surgeon (ES) conducted all oral evaluations and maximum mouth opening (MMO) measurements. RIT was defined as an MMO threshold of 35 mm or less, in line with criteria established by Dijkstra et al. [4]. We used Therabite^®^ (Atos Medical AB, Hörby, Sweden) for validated, disposable, and easily used features, to measure the MMO [14]. Patients were instructed to expand their mouths as much as possible while wearing the Therabite^®^ motion scale to measure the distance between the lower edge of one upper central incisor and the matching upper edge of one mandibular central incisor. The mean MMO was calculated by averaging three consecutive measurements during each session. To assess RIT, we measured MMO for each patient at 1, 3, 6, 9, and 12 months following CCRT. Additional measurements were taken as needed and during subsequent scheduled appointments.

### Calculation of Global Immune-Nutrition-Inflammation Index (GINI)

The GINI was calculated using the original formula [12]: *GINI ═ (CRP × M × P × N) ÷ (Albumin × L),* where CRP, M, P, N, and N correspond to the counts of CRP, monocytes, platelets, neutrophils, lymphocyte, and albumin measures read prior to the administration of the initial CCRT dose.

### Ethical statement

The retrospective research design used in the present study (project No: KA23/196) received approval from the institutional review board at Baskent University Medical Faculty. The research followed the Guidelines for Good Clinical Practice and the Declaration of Helsinki, including any future revisions regarding its principles and standards. Before undergoing CCRT, all patients were required to provide their signed informed consent, which permitted researchers to examine their clinical and blood test data and publish any pertinent discoveries. Informed consent forms were obtained from all patients before commencing the evaluation process to obtain and analyze the patients’ sociodemographic, dental, and medical records and blood samples and disseminate the outcomes in academic societies. Each participant has provided written informed consent for collecting, analyzing, and disseminating their results.

### Statistical analysis

The primary objective of this investigation was to analyze the correlation between pre-CCRT GINI measures and post-CCRT RIT rates. We employed percentage frequency distributions to quantify categorical variables, while medians and ranges were utilized to explicate continuous variables. The Student’s *t*-test, the chi-square test, or Spearman correlation analysis were executed for intergroup comparisons. These analytical methods were chosen based on their appropriateness for the data set and statistical objectives. The current study utilized receiver operating characteristic (ROC) curve analysis, a widely recognized robust and valid statistical tool for evaluating the diagnostic accuracy of a test or a biomarker, to estimate the optimal pre-CCRT cutoffs that can effectively stratify the research cohort into two GINI groups with significantly distinct outcomes. All comparisons were two-sided and a *P* value less than 0.05 was deemed significant. The univariate analysis included all the factors exhibiting significance in the association between post-CRT RIT rates and the baseline or treatment-related characteristics, while the multivariate logistic regression analysis included only the factors exhibiting significance or a strong trend toward significance (*P* < 0.10) in the univariate analysis.

## Results

### Patient characteristics and treatment modalities

The present study included 230 LA-NPC patients who fulfilled the eligibility criteria. As shown in Table 1, the median age was 56 years (range: 18–76 years) for the whole cohort, with 158 (68.7%) male patients. The rates of tobacco and alcohol consumption were 63.5% and 32.2%, respectively. Most patients had advanced disease stages: 171 (73.5%) had T3–4, and 183 (79.6%) had N2–3 disease stages. The final post-CCRT MMO measurements demonstrated a median reduction of 3.2 mm (7.73%; range: 1.4%–39.7%) from a pre-CCRT median of 41.4 mm (range: 37.4–46.8 mm) to a final median of 38.2 mm (range: 25.9–44.0 mm) (Tables 1 and 2). During the follow-up period, 52 patients (22.6%) received RIT diagnosis based on Dijkstra’s trismus criteria for cancer patients (MMO ≤ 35 mm) [3], with a median CCRT to RIT duration of ten months (range: 6–18 months).

**Table 1:** Baseline and treatment characteristics of the whole study cohort per Global Immune-Nutrition-Inflammation Index group

**Characteristics**	**All patients (*N* ═ 230)**	**GINI-1 (**<**1424) (*N* ═ 140)**	**GINI-2 (**≥**1424) (*N* ═ 90)**	***P* value**
Median age (years)	56 (18–76)	56 (20–76)	57.5 (18–76)	0.06
*Age group, N (%)*				
>56 years	112 (48.7)	66 (49.1)	46 (48.9)	0.6
≤56 years	118 (51.3)	74 (52.9)	44 (51.1)	
*Gender, N (%)*				
Male	158 (68.7)	96 (68.6)	62 (68.9)	1.0
Female	72 (31.3)	44 (31.4)	28 (31.1)	
*Smoking status, N (%)*				
Yes	146 (63.5)	90 (64.3)	56 (62.2)	0.8
No	84 (36.5)	50 (35.7)	34 (37.8)	
*Alcohol consumption, N (%)*				
Yes	74 (32.2)	43 (30.7)	31 (34.4)	0.6
No	156 (67.8)	97 (69.3)	59 (65.6)	
Median pre-CCRT MMO, mm (range)	41.4 (37.4–46.8)	41.6 (38.4–46.8)	41.0 (37.4–45.0)	0.2
*Pre-C-CRT MMO group, N (%)*				
<41.4 mm	115 (50)	65 (46.4)	50 (55.6)	0.22
≥41.4 mm	115 (50)	75 (53.6)	40 (44.4)	
*T-stage, N (%)*				
1-2	59 (25.7)	38 (27.1)	21 (23.3)	0.54
3-4	171 (74.3)	102 (72.9)	69 (76.7)	
*N-stage, N (%)*				
0-1	47 (20.4)	29 (20.7)	18 (20.0)	0.89
2-3	183 (79.6)	111 (79.3)	72 (80.0)	

GINI: Global Immune-Nutrition-Inflammation Index; pre: Pretreatment; C-CRT: Concurrent chemoradiotherapy; MMO: Maximum mouth opening; mm: Millimeter; T: Tumor; N: Node.

**Table 2:** Treatment characteristics of the entire study cohort per Global Immune-Nutrition-Inflammation Index Group

**Characteristics**	**All patients (*N* ═ 230)**	**GINI-1 (<1424) (*N* ═ 140)**	**GINI-2 (≥1424) (*N* ═ 90)**	***P* value**
Mean MAD, Gy (range)	37.2 (11.9–65.3)	38.3 (12.5–64.3)	36.7 (11.9–65.3)	0.46
*Mean MAD group, N (%)*				
<37.2 Gy	119 (51.7)	74 (52.9)	45 (50.0)	0.67
≥37.2 Gy	111 (48.3)	66 (47.1)	45 (50.0)	
*MAD V53.2 Gy group, N (%)*				
<38.6%	98 (42.6)	58 (41.4)	40 (44.4)	0.81
≥38.6%	132 (57.4)	82 (58.6)	50 (55.6)	
Median time from CCRT to RIT, months (range)	10 (6–18)	10 (7–13)	10 (6–18)	0.77
Median post-CCRT MMO, mm (range)	38.2 (25.9–44.0)	39.0 (28.3–44.0)	36.5 (25.9–43.2)	<0.001
*Concurrent chemotherapy cycles, N (%)*				
1	51 (22.2)	32 (22.9)	19 (21.1)	0.73
2–3	179 (77.8)	108 (77.1)	71 (78.9)	
*Adjuvant chemotherapy cycles, N (%)*				
0	59 (25.7)	38 (27.1)	21 (23.3)	0.39
1–2	171 (74.3)	102 (72.9)	69 (76.7)	
*Post-CCRT RIT, N (%)*				
Absent	178 (77.4)	127 (90.7)	51 (56.7)	<0.001
Present	52 (22.6)	13 (9.3)	39 (43.3)	

GINI: Global Immune-Nutrition-Inflammation Index; MAD: Masticatory apparatus dose; Gy: Gray; V: Volume; post: Postoperative; CCRT: Concurrent chemoradiotherapy; mm: Millimeter; RIT: Radiation-induced trismus.

**Figure 1.**
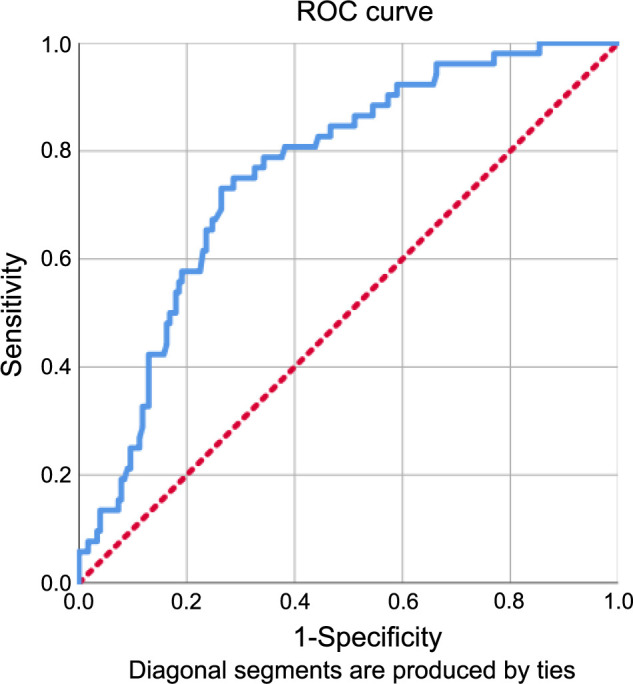
**The outcomes of a receiver operating characteristic curve analysis examining the correlation between the GINI and RIT rates (GINI cutoff: 1424; Area under the curve: 76%; sensitivity: 75.0%; specificity: 71.7%, J-index: 0.463).** GINI: Global Immune-Nutrition-Inflammation Index; RIT: Radiation-induced trismus; ROC: Receiver operating characteristic.

In the present study, we conducted an ROC curve analysis to investigate the potential correlation between the incidence rates of RIT and the baseline GINI levels. The analysis revealed that a meaningful relationship could be observed at a cut-off of 1424 [Area under the curve (AUC): 76%; sensitivity: 75.0%; specificity: 71.7%, J-index: 0.463] (Figure 1). Therefore, we divided the study population into two groups: Group 1 (GINI < 1424; *N* ═ 140) and Group 2 (GINI ≥ 1424; *N* ═ 90). The chi-square test was then used to compare RIT incidence between the two groups, further indicating a strong correlation between higher baseline GINI levels and increased post-CCRT RIT rates. Specifically, the incidence of RIT was significantly higher in Group 2 (GINI ≥ 1424) compared to Group 1 (GINI < 1424) [43.3% vs. 9.3%; hazard ratio (HR): 4.76; *P* < 0.001].

### The results of univariate and multivariate analysis

**Figure 2.**
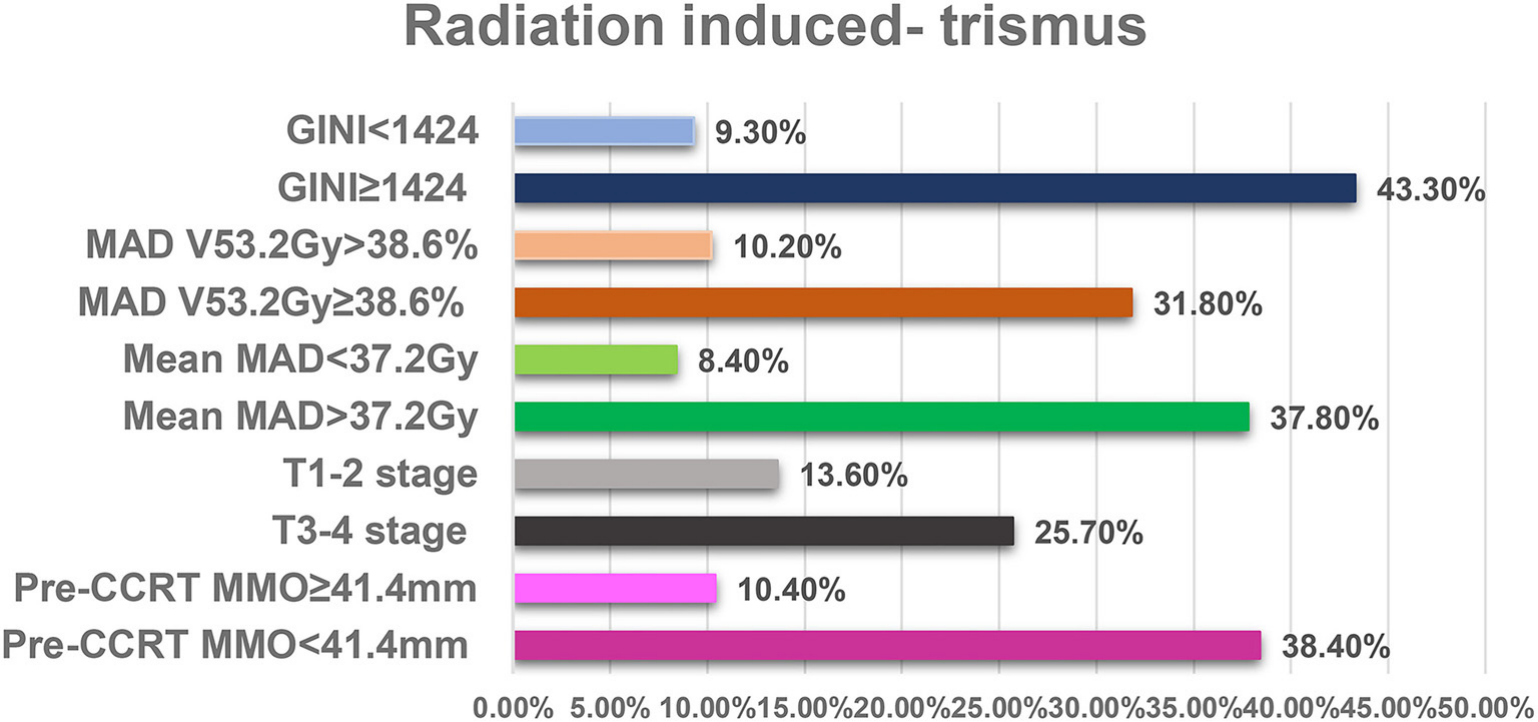
**Bar graph displaying the frequency of radiation-induced trismus based on significant variables in multivariate analyses**. MAD: Mandibular apparatus dose; T: Tumor; V: Volume; CCRT: Concurrent chemoradiotherapy; GINI: Global Immune-Nutrition-Inflammation Index; RIT: Radiation-induced trismus; MMO: Maximum mouth opening.

The outcomes of univariate analyses revealed a significant association between elevated RIT incidences and certain patients groups as shown in Table 3: median MMO < 41.4 mm (38.4% vs. 10.4% for ≥ 41.4 mm; *P* < 0.001), T3-4 tumor stage (25.7% vs. 13.6% for T1-2; *P* ═ 0.009), pre-CCRT GINI measures ≥ 1424 (43.3% vs 9.3% for < 1424, *P* < 0.001), mean masticatory apparatus dose (MAD) ≥37.2 Gy (37.8% vs 8.4% for mean MAD < 37.2), *P* < 0.001), MAD V53.2 Gy ≤ 38.6% (31.8% vs. 10.2% for V53.2 > 38.6%; *P* < 0.001). Although the female gender showed a trend for elevated RIT rates (29.2% vs 19.6% for the male gender; *P* ═ 0.072), the difference did not achieve statistical significance (*P* ═ 0.072). Further analysis using multivariate logistic regression, including these six factors, revealed that all factors, except for female gender, were independent and significant predictors of RIT in patients with LA-NPC who underwent definitive CCRT (*P* < 0.05 for each), as shown in Table 3 and Figure 2.

**Table 3:** Radiation-induced trismus outcomes of the entire study group

**Characteristics**	**All patients (*N* ═ 230)**	**RIT (%) (*N* ═ 52)**	**Univariate *P* value**	**Multivariate *P* value**	**HR (95% CI)**
*Age group, years, N (%)*					
≤56	112 (48.7)	27 (24.1)	0.64	–	–
>56	118 (51.3)	25 (21.1)			
*Gender, N (%)*					
Male	158 (68.7)	31 (19.6)	0.042	0.072	1.42 (0.96–2.14)
Female	72 (31.3)	21 (29.2)			
*Smoking status, N (%)*					
Yes	146 (63.5)	32 (21.9)	0.55	–	–
No	84 (36.5)	20 (23.8)			
*Alcohol consumption, N (%)*					
Yes	74 (32.2)	13 (17.6)	0.24	–	–
No	156 (67.8)	39 (25.0)			
*Pre-CCRT MMO group, N (%)*					
<41.4 mm	115 (50)	40 (34.8)	<0.001	<0.001	3.74 (2.12–5.81)
≥41.4 mm	115 (50)	12 (10.4)			
*T-stage group, N (%)*					
1-2	59 (25.7)	8 (13.6)	0.007	0.009	1.73 (1.18–3.07)
3-4	171 (74.3)	44 (25.7)			
*N-stage group, N (%)*					
0-1	47 (20.4)	10 (21.3)	0.74	–	–
2-3	183 (79.6)	42 (23.0)			
*Pre-CCRT GINI group, N (%)*					
<1424	140 (60.9)	13 (9.3)	<0.001	<0.001	4.94 (2.07–7.81)
≥1424	90 (39.1)	39 (43.3)			
*Concurrent chemotherapy cycles, N (%)*					
1	51 (22.2)	10 (19.6)	0.49	–	–
2-3	179 (77.8)	42 (23.5)			
*Adjuvant chemotherapy cycles, N (%)*					
0	59 (25.7)	13 (22.0)	0.88	–	–
1-2	171 (74.3)	39 (22.8)			
*Mean MAD group, N (%)*					
<37.2 Gy	119 (51.7)	10 (8.4)	<0.001	<0.001	4.76 (2.38–6.87)
≥37.2 Gy	111 (48.3)	42 (37.8)			
*MAD V53.2 Gy group, N (%)*					
<38.6%	98 (42.6)	10 (10.2)	<0.001	<0.001	2.97 (1.91–4.86)
≥38.6%	132 (57.4)	42 (31.8)			

GINI: Global Immune-Nutrition-Inflammation Index; HR: Hazard ratio; RIT: Radiation-induced trismus; pre: Pretreatment; C-CRT: Concurrent chemoradiotherapy; MMO: Maximum mouth opening; mm: Millimeter; T: Tumor; N: Node; MAD: Masticatory apparatus dose; Gy: Gray; V: Volume.

## Discussion

This retrospective study aimed to assess the usefulness of pre-CCRT GINI levels as a predictor of RIT rates in LA-NPC patients. Our most notable finding was that patients with a pre-CCRT GINI ≥ 1.424 had a significantly higher RIT incidence compared to those with a GINI < 1.424 [43.3% vs 9.3%; HR: 4.76; *P* < 0.001]. Other significant findings included a strong correlation between increased RIT rates and pre-CCRT MMO < 41.4 mm (*P* < 0.001), T3–4 stage (*P* ═ 0.009), mean MAD > 37.2 Gy (*P* < 0.001), and MAD V53.2 Gy ≥ 38.6% (*P* < 0.001).

We have examined several factors that may be associated with RIT. Among them, MMO < 41.4 mm (*P* < 0.001), T3–4 disease stage (*P* < 0.001), and MAD V53.2 ≥ 38.6% (*P* < 0.001) have shown significant associations with RIT rates. Several previous studies have investigated the effects of pre-CCRT MMO and MAD on RIT rates in patient groups similar to our current cohort. However, the number of studies conducted is limited, and various MMO cutoffs have been proposed including <46 mm [15], <40 mm [16], and ≤40.7 mm [9]. Recently, Somay et al. [10] determined 41.4 mm as the optimal MMO cutoff for LA-NPC patients, which is quite similar to our finding of 41.6 mm. The common thread among these studies is that they demonstrate that patients who are close to or below the lower limit of the normal MMO range (40–60 mm) before RT or CCRT are more likely to experience RIT after treatment [17]. In a study by Dworkin et al., an MMO of less than 40 mm was associated with mastication muscle disorders. While this value may not apply to all individuals, a measure below this critical MMO level has been reported in individuals without TMD due to parafunctional habits like bruxism [17, 18]. Therefore, patients with MMO measures close to the definition of RIT (<35 mm) may have a greater chance of experiencing RIT due to the consequent effects of radiation-induced fibrosis in the masticatory muscles, synovial fluid, and TMJ. Given that even a small reduction in MMO width may push their measures below the recognized RIT cutoff, it is important to closely monitor and promptly address patients with MMO < 40–45 mm to prevent or treat RIT.

Various researchers have identified a wide variety of additional patient, disease, clinical, and dosimetric factors that contribute to the occurrence of RIT [19, 20]. Among these, a crucial variable is the size or the volume of the tumor, which leads to the unavoidable irradiation of neighboring tissues with higher doses of radiation. Larger radiation volumes are mandatorily used in more advanced malignancies, namely T3–4 LA-NPCs, which, unfortunately, expose the masticatory apparatus components to higher RT doses [21]. Higher radiation doses to the larger volumes of masticatory muscles, TMJ, and associated ligaments may induce aggravated and persistent local and systemic inflammation, vascular occlusion, tissue hypoxia, and ultimately, tissue fibrosis, which are more prominent in higher T-stage NPCs due to the dose-volume relationship between the irradiated volume size and the probability of radiation-induced toxicities, including RIT [5]. While there may be additional pathophysiological mechanisms at play, the information presented provides a rational explanation for the potential association between the advanced T-stages and increased rates of RIT (25.7% for T3-4 vs 13.6% for T1-2; *P* ═ 0.009) observed in this study and its predecessors.

Although there is limited research on MAD, studies have shown that the dose received by the components of the masticatory apparatus, measured as a dosimetric parameter for 100% volume, is associated with RIT rates [8, 9]. Our research findings indicate that RIT is significantly more likely to occur when the mean MAD is > 37.2 Gy (*P* < 0.001) and MAD V53.2 Gy is ≥ 38.6% (*P* < 0.001). These findings align with a retrospective study conducted by Somay et al. [9], which also examined the impact of MAD on RIT rates in 198 patients who received RT for LA-NPCs, which determined the critical dose as a mean MAD >57.2 Gy. These results suggest that the mean MAD and MAD Vx% effectively measure the level of damage in the masticatory apparatus, which functions as a parallel organ. The mean dose captures the extent of damage occurring throughout the entire organ, while MAD Vx% quantifies the volume of the affected organ, namely the masticatory apparatus in this scenario. Therefore, while there is currently a lack of information from more extensive studies to provide strict recommendations for exact threshold doses in routine RT planning processes, it is still advisable to include mean MAD and MAD Vx% values as appropriate metrics in dose planning. We believe this approach can effectively reduce masticatory apparatus dosages, thereby mitigating the risk of RIT without compromising the efficacy of tumor control rates if RT plans are executed carefully.

Our analysis made a unique and vital addition to the literature on LA-NPC by finding pre-CCRT GINI values as a robust predictor of RIT rates. In this respect, we discovered that patients with a pre-CCRT ≥ 1424 had significantly higher RIT rates than their comparators with a GINI < 1424 [43.3% vs 9.3%; HR: 4.76; *P* < 0.001]. Although assigning this finding to a single reason without relevant data is challenging, we can infer some plausible mechanisms by dividing GINI into separate components. Among many others, one reasonable approach is to reformulate GINI as PIV × CRP-to-Albumin Ratio (CAR), which denotes the combination of a cellular and a biochemical biological marker. Somay et al. [10] recently conducted a study on 223 patients with LA-NPC. The study sought to identify the optimal cut-off point for PIV. The research results indicated 850 as the optimal PIV cut-off, and RIT rates were significantly higher in the PIV > 850 group than its PIV ≤ 830 counterparts (60.3% vs 5.0%; OR: 5.79; *P* < 0.001). This effect is most likely associated with the combined effect of the imbalanced counts of platelets, monocytes, neutrophils, and lymphocytes [PIV ═ (P×M×N) ÷ L] [10], which directly or indirectly aggravates the inflammatory, hypoxic, hypovascular, and fibrotic pathogenesis and progression of RIT [8, 9, 22, 23].

The impact of pre-CCRT CAR measures on the rates of RIT has yet to be studied, rendering our data impossible to compare. It is well established that RT causes an imbalance in cytokine levels, leading to a persistent inflammatory response and, thus, the increased production of acute-phase proteins such as CRP, a component of the CAR formula [24]. CRP serves as a reliable systemic inflammation marker, while albumin is a well-known indicator of leanness and nutrition. Consequently, related studies have shown that the CAR is a valuable metric for evaluating systemic inflammation and nutritional status [25, 26]. Increased CRP levels are consistently associated with decreased hepatocyte albumin synthesis. This reduction leads to an elevation in the measure of the CAR, regardless of the underlying cause. Therefore, it is imperative to consider the CAR as a marker of systemic inflammation in various clinical settings, as it is a reliable indicator of the inflammatory response in the body. Elevated levels of CRP and associated CAR can stimulate macrophages to produce tissue factor, a potent procoagulant that can result in increased intravascular coagulation and thrombosis during inflammatory states [27]. This process can lead to a state of tissue hypoxia, thereby exacerbating existing systemic and local inflammation. Increased CRP levels facilitate tissue fibrosis in different disease states by initiating TGF-β/Smad signaling via both TGF-β1-dependent and TGF-β1-independent pathways [27]. Unfortunately, no previous research exists examining CAR’s predictive power in determining the RIT rates of HNC patients treated with RT or CCRT. Yet, in light of the cumulative information, the GINI index appears to be a comprehensive measure encompassing all components of the cellular PIV and biochemical CAR indices. Therefore, GINI integrates six factors, four cellular and two biochemical, rendering it a holistic novel biomarker compared to PIV, CAR, or their fragmented blends. Each cellular or biochemical component of the GINI index plays a crucial role in every step of the initiation, development, and progression of RIT, including vascular occlusion, hypoxia, hyperinflammation, and hyperfibrosis, which are characteristic hallmarks of RIT pathogenesis. Although further research is needed to confirm our findings, the pre-CCRT GINI index appears to be a novel and robust biological marker that reliably categorizes LA-NPC patients into two RIT risk groups.

This study has certain limitations. Firstly, it was a retrospective study conducted by a single institution, which may have led to biases commonly found in this type of research. Secondly, we did not investigate any potential correlations between GINI groups and other biomarkers, such as cytokines and chemokines produced and released by specific GINI components. Lastly, our findings were based on a single instance of GINI data before CCRT, which may affect the accuracy of our results as GINI levels can vary significantly during and after C-CRT due to changes in tumor burden, inflammatory status, and host immunity. Therefore, further research is needed to establish a definitive connection between pretreatment GINI levels and RIT rates.

## Conclusion

The objective of this study was to examine if pre-treatment GINI values can predict RIT in patients with LA-NPC who undergo definitive CCRT. Our results demonstrate that this comprehensive index can accurately divide patients into two distinct RIT groups. However, given the study’s retrospective and single-center design, additional research is necessary to confirm these findings before promoting this index as a new stratification tool for RIT in patients with LA-NPC receiving definitive CCRT.

## Data Availability

The data supporting this study’s findings is available from the corresponding author, Efsun Somay, upon a reasonable special request.
